# Late-onset anastomotic leak following sweet esophagectomy: A case report and review of the literature

**DOI:** 10.1097/MD.0000000000022479

**Published:** 2020-10-02

**Authors:** Feng-Wei Kong, Wei-Min Wang, Lei Liu, Wen-Bin Wu, Long-Bo Gong, Miao Zhang

**Affiliations:** a Department of General Surgery, Xuzhou Infectious Disease Hospital, Xuzhou; b Department of Gastroenterology of Yichang Central People's Hospital, Institute of Digestive Disease, China Three Gorges University, Yichang; c Department of Surgery, Xuzhou Central Hospital, Xuzhou, China.

**Keywords:** anastomotic leakage, esophagectomy/oesophagectomy, serratus anterior plane block

## Abstract

**Rationale:**

Late-onset anastomotic leak (AL) is an uncommon but potentially lethal complication after esophagectomy.

**Patient concerns:**

A 74-year-old male patient was readmitted due to chest distress and chills about 3 months after initial esophagectomy for cancer.

**Diagnoses:**

The previous endoscopic biopsy revealed primary esophageal squamous cell carcinoma, and sweet esophagectomy with gastric conduit reconstruction was therefore performed. The patient developed AL 3 months after the surgery.

**Interventions:**

Naso-leakage extraluminal drainage tube was utilized because the symptoms of the patient were aggravated 1 month after the chest tube drainage since his second admission for AL.

**Outcomes:**

Twenty-one days after naso-leakage extraluminal drainage, the computed tomography images showed the healing of the leakage. Then the patient was discharged from the hospital.

**Lessons:**

Late-onset AL should be kept in mind when the patient complained of chest distress and fever during the follow up after esophagectomy. In addition, naso-leakage extraluminal drainage could be considered for the treatment of AL. Further trials for better evidence are warranted.

## 1 Introduction

It is reported that about 38.1% of the patients experienced ≥ 1 complications within 30 days following esophagectomy; whereas 10.7% of them experienced unplanned readmissions due to inflammation and pulmonary/gastrointestinal complications.^[1]^ Moreover, the pulmonary complications and anastomotic leakage (AL) might result in decreased long-term survival of the patients after esophagectomy.^[2]^ Furthermore, AL is associated with increased morbidity and mortality due to mediastinitis and thoracic contamination. The drainage techniques such as endoscopic vacuum therapy and pigtail are the first-line therapeutic options for gastrointestinal transmural defects.^[3]^

AL usually occurs within 10 days after esophagectomy, but in some cases, it may occur as late as a few weeks after surgical resection of the esophagus.^[4]^ Herein we presented a patient who developed AL 3 months after Sweet esophagectomy, followed by a brief literature review.

## 2 Case presentation

A 74-year-old male non-smoker was admitted because of the gradually aggravated dysphagia and fatigue for nearly 2 months. Laboratory tests and radiographic exams were conducted as thorough physical examination showed nothing abnormal. The serum tumor biomarkers such as carcinoembryonic antigen, neuron-specific enolase, cytokeratin-19 fragment, and carbohydrate antigen 125 were all in normal range. Contrast-enhanced computed tomography (CT) revealed the thickened lower-third esophageal wall; whereas there were no obviously enlarged supraclavicular lymph nodes or distant metastasis. Fine-needle biopsy in July 2017 revealed the pathological diagnosis of primary esophageal squamous cell cancer.

After the multi-disciplinary consultation and a strict preoperative workup, Sweet esophagectomy and systemic mediastinal lymphadenectomy was scheduled. The operation was performed successfully under general anesthesia. R0 resection was achieved. The operation time was 150 minutes; while the estimated intraoperative blood loss was 400 mL. Jejunostomy for enteral nutrition and a nasogastric tube for gastric decompression were routinely used. Furthermore, ultrasound-guided serratus anterior plane block using bupivacaine liposome was utilized for postoperative analgesia. Moderately differentiated esophageal squamous cell cancer (pT1bN0M0G2, IB) was confirmed.^[5]^

**Figure 1:**
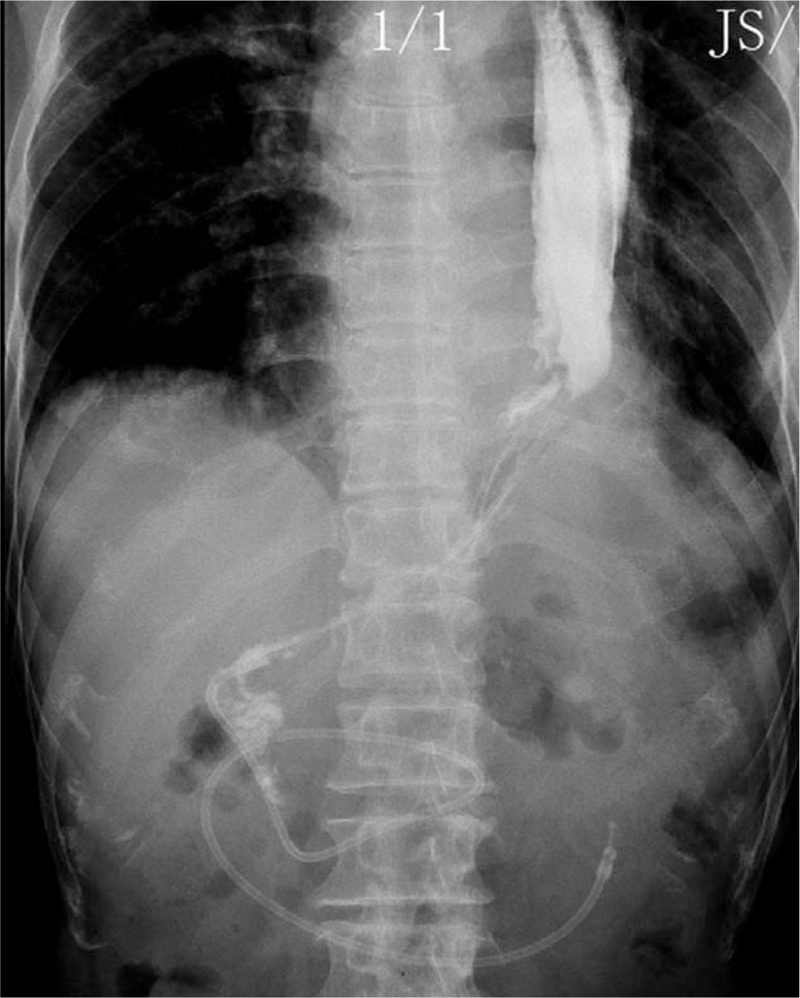
Postoperative esophagography excluded the anastomotic leak.

Besides tube feeding, the patient started drinking clean water on postoperative day (POD) 3. Moreover, normal oral diet was initiated on POD 6 as esophageal or gastric leakage was excluded (Fig. 1). The patient was discharged from the hospital on POD 7 uneventfully. Adjuvant treatment was not applicable.

**Figure 2:**
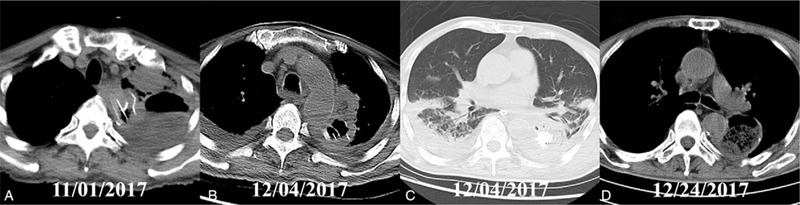
The radiographic images of the patient. (A) The CT on his readmission showed left-sided pleural effusion, in suspicious of late-onset anastomotic leakage. (B) Bilateral aspiration pneumonia was indicated 1 month after the chest tube drainage. (C) Left-sided atelectasis and residual pleural effusion after 1 month of chest tube drainage. (D) The pulmonary field turned to be clean after 20 days of naso-leakage extraluminal drainage.

Three months after the surgery, the patient was readmitted in November 2017 due to fatigue, dyspnea, productive cough, and chills. Respiratory infection was initially diagnosed empirically; however, these symptoms were gradually aggravated 3 days after the administration of intravenous piperacillin/sulbactam (3.0 g, twice daily). Bacteria, fungus, or tuberculosis were not detected by repeated sputum cultures. CT was therefore conducted, which indicated left-sided pleural effusion (Fig. 2A). Based on the above findings, late-onset AL was strongly suspected.

A 28 French catheter was reinserted for chest drainage. Then AL was confirmed by the return of turbid yellow fluid from the thorax. Total enteral nutrition through a nasojejunal tube was administered, along with broad-spectrum antibiotics. Nevertheless, bilateral aspiration pneumonia, and left-sided atelectasis emerged 1 month after the treatment in December 2017 (Figs. 2B and 2C).

After another multi-disciplinary consultation, naso-leakage extraluminal drainage was performed as reported.^[6]^ In detail, a multifunctional 12 French tube was placed through the leakage to the bottom of vomica.^[7]^ When the vomica diminished, the tube could be pulled out gradually. Thereafter, the symptom of this patient was gradually alleviated. Encouragingly, the healing of the AL was confirmed about 20 days later in the CT images (Fig. 2D). During the follow up of 2 years, the patient demonstrated satisfactory quality of life without tumor recurrence or metastasis.

## 3 Discussion

A timely diagnosis and effective management are essential to avoid AL-related problems; however, an early diagnosis of AL might be challenging for lacking of reliable biomarkers. It is reported that the serum C-reactive protein and leucocytes, as well as amylase in peritoneal drain are insufficient as predictive biomarkers of AL.^[8]^ Oral contrast studies have low sensitivity in detecting ALs, which can also lead to unnecessary prolonged hospital stay after surgery.^[9]^ There are several issued could be elucidated accordingly.

To our knowledge, late-onset AL after esophagectomy is rare. A total of 9 reports in terms of late-onset AL has been reviewed before.^[9]^ In case of suspicion of leakage, the chest/abdomen CT with oral contrast agent or endoscopy always need to be performed.^[10]^ The estimated incidence of delayed AL after esophagectomy is about 2.8% (1.8%-4.4%), as most of them occurred within 4 to 6 weeks after the surgery.[^11^,^12^,^13^,^14^,^15^,^16^,^17^,^18^,^19^]

Anastomotic drainage facilitates earlier identification and resolution of the leaks. Moreover, anastomosis reinforcement with omentoplasty may effectively lower the incidence of AL.^[20]^ Conservative approaches including endovascular clips or stents, intraluminal endoscopic vacuum therapy, self-expandable metal stent with a silk thread, and a percutaneous endoscopic gastrostomy tube have been reported to be useful as initial management in treating AL.[^21^,^22^] However, surgical intervention is sometimes required for refractory mediastinal contamination. No evidence supporting a specific treatment option for post-esophagectomy AL has been obtained for lacking of high-quality studies.[^23^,^24^,^25^,^26^,^27^]

The naso-leakage extraluminal drainage has been reported to be effective in the treatment of AL.^[6]^ For the present patient, the AL was healed using naso-esophageal extraluminal drainage without negative pressure device. The previous reports regarding AL treated by naso-esophageal extraluminal drainage was summarized in Table 1. Based on the currently available evidence, naso-leakage extraluminal drainage might be considered as the first-line treatment for esophageal leakage and perforations. Similarly, cervical end-esophageal exteriorization in severe intrathoracic AL also resulted in rapid control of the inflammation.^[28]^ It is noteworthy that an updated guideline or consensus recommendation for the treatment of AL is warmly welcomed, which should aim to decrease the risk of AL-related severe contamination such as the potentially lethal systemic inflammatory response syndrome.

**Table 1:**
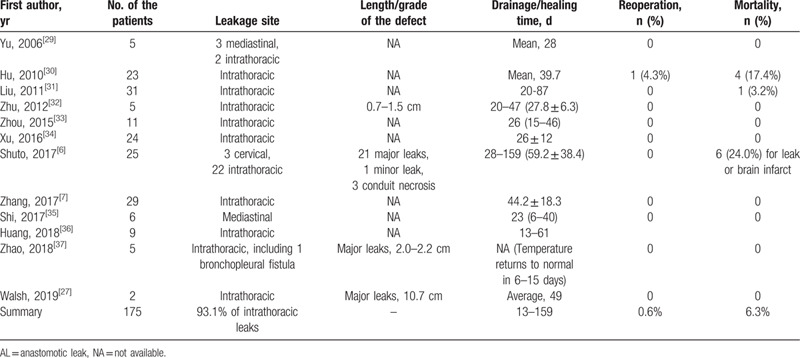
Previous reports of naso-leakage extraluminal drainage for anastomotic leakage after esophagectomy.

## 4 Conclusions

Late onset AL should be kept in mind when the patient complains of chest distress or persistent fever after esophagectomy. Naso-leakage extraluminal drainage is effective for the treatment of AL. However, better evidence is still needed regarding the optimal therapeutic option for post-esophagectomy intrathoracic AL.

## Author contributions

**Conceptualization:** Feng-Wei Kong, Wei-Min Wang.

**Data curation:** Wei-Min Wang.

**Funding acquisition:** Wen-Bin Wu.

**Methodology:** Lei Liu, Long-Bo Gong.

**Resources:** Miao Zhang.

**Writing – original draft:** Feng-Wei Kong, Wen-Bin Wu, Long-Bo Gong.

**Writing – review & editing:** Miao Zhang, Lei Liu, Wen-Bin Wu.
